# Breast Cancer Nodes Detection Using Ultrasonic Microscale Subarrayed MIMO RADAR

**DOI:** 10.1155/2014/797013

**Published:** 2014-09-15

**Authors:** Attaphongse Taparugssanagorn, Siwaruk Siwamogsatham, Carlos Pomalaza-Ráez

**Affiliations:** ^1^Wireless Information Security and Eco-Electronics Research Unit, National Electronics and Computer Technology Center, 112 Phahon Yothin Road, Klong 1, Klong Luang, Pathum Thani 12120, Thailand; ^2^College of Engineering, Technology, and Computer Science, Department of Engineering, Purdue University, Fort Wayne, IN 46805, USA

## Abstract

This paper proposes the use of ultrasonic microscale subarrayed MIMO RADARs to estimate the position of breast cancer nodes. The transmit and receive antenna arrays are divided into subarrays. In order to increase the signal diversity each subarray is assigned a different waveform from an orthogonal set. High-frequency ultrasonic transducers are used since a breast is considered to be a superficial structure. Closed form expressions for the optimal Neyman-Pearson detector are derived. The combination of the waveform diversity present in the subarrayed deployment and traditional phased-array RADAR techniques provides promising results.

## 1. Introduction

Breast cancer is the most common cancer among women in many countries in the world. Early detection of this type of cancer increases the likelihood of a successful treatment. X-Ray mammography is considered the most effective imaging technology to detect early-stage breast cancer [1]. In spite of its wide use, mammography does not provide accurate results. On average 20% of false-negative results occur when mammograms appear normal even though cancer is present. These results are due mainly to high breast density [2]. When mammograms are used over a period of time, for example, once a year for ten years, the percentage of having a false-positive result can be as high as 50%. When a false-positive result occurs additional tests are needed, for example, a biopsy to determine whether a cancer is present. False-positive results can also lead to anxiety and other forms of psychological distress in the affected women [3]. As an alternative technology, microwave imaging has been proposed since at microwave frequencies there is a significant difference in the dielectric properties of normal and malignant breast tissue [4–6]. Furthermore, the attenuation of microwave signals in a normal breast tissue is low enough to facilitate their propagation through even a large breast volume. Microwave technology provides high contrast but it lacks potential for high spatial resolution. Electromagnetic waves at lower frequencies than microwaves suffer from poor propagation within body tissues leading to high attenuation levels. Another factor to take into account is the in-body transmissions which have to be low-power constrained to prevent overheating of tissues and the consequent death of cells.

Magnetic resonance imaging (MRI) scans use pulses of radio waves and strong magnets to provide detailed images of body parts [7, 8]. However for MRI to be effective in detecting breast cancer a contrast liquid called gadolinium needs to be injected into a vein so the scan can show good details. This process takes a long time, up to an hour, and it is expensive.

Another option to consider is the use of echoes from ultrasound waves impinging on parts of the breast. In [9] the feasibility of using ultrasound waves for communications in intrabody area networks was discussed. That study included the fundamentals of ultrasonic propagation in human tissues and explored the choices proper transmission frequency, transmission power, bandwidth, and transducer size. An important advantage of using ultrasound waves is that the tests are painless and do not expose the body to radiations. Based on the medical experience of several decades ultrasound is safe, with no dangerous bioeffects being observed, as long as the energy delivered to the tissues is less than 50 J/cm^2^ [10]. Also, ultrasound technology might be the most helpful for those women with high density breasts.

For the general problem of target position detection and estimation, multiple input multiple output (MIMO) RADAR is an attractive option to increase the quality of the results. Unlike a standard phased-array RADAR which transmits scaled versions of a single waveform [11, 12], a MIMO RADAR system can use multiple different probing signals that can be chosen quite freely [13]. This diversity in the type of signals can provide better results when compared with a standard phased array RADAR. A MIMO RADAR system consists of collocated transmit and receive antennas for which it has been shown to offer high resolution [14] and high sensitivity to detect slowly moving targets [15]. MIMO RADAR also offer the ability to have waveform optimization.

Due to the advantages of ultrasound and MIMO RADAR systems this paper proposes a noninvasive method comprised of an ultrasonic, microscaled, collocated, and subarrayed MIMO RADAR for the high resolution detection of multiple breast cancer nodes of less than 1 centimeter in size. The results show that a subarrayed deployment in combination with traditional phased-array RADAR and optimal Neyman-Pearson detection provides very promising results.

The rest of the paper is organized as follows.  Section 2 formulates the problem including a signal model, that is, the microscaled subarrayed MIMO RADAR and the propagation model.  Section 3 describes the analytical solution of the multiple target detection problem.  Section 4 has the simulation results. The conclusions are given in Section 5.

## 2. System Model

An ultrasound is a mechanical wave, similar in nature to an audible sound, but at frequencies greater than 20 kHz. Medical ultrasound devices use ultrasound waves in the range of 1–20 MHz. The selection of a proper transducer frequency is a very important factor to obtain an optimal image resolution. High-frequency ultrasound waves (short wavelength) generate images of high axial resolution. However, for a given distance high-frequency waves are more attenuated than lower frequency waves; thus, they are more suitable for imaging of superficial structures. Conversely, low-frequency waves (long wavelength) provide images of lower resolution but can penetrate into deeper structures due to a lower degree of attenuation. For this reason, it is preferred to use high-frequency transducers (up to 10–15 MHz range) to image superficial structures and to use low-frequency transducers (typically 2–5 MHz) for imaging deep structures, for example, lumbar neuraxial ultrasound. A breast is considered to be a superficial structure and, therefore, high-frequency waves are the preferred choice.

**Figure 1:**
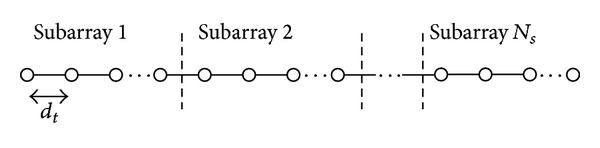
Subarrayed MIMO RADAR transmit architecture.

**Figure 2:**
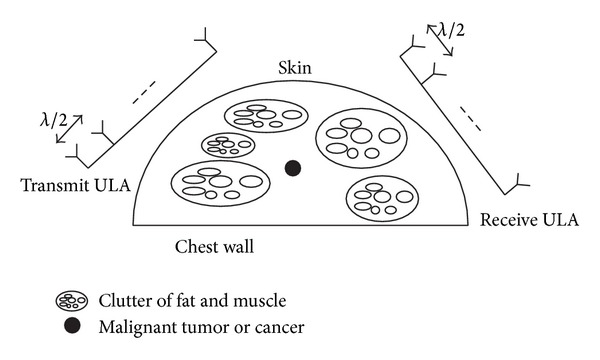
Breast model and MIMO RADAR placement.

A microscaled subarrayed MIMO RADAR system with *N*_*t*_ transmit antennas and *N*_*r*_ receive antennas is considered. Both transmit and receive antenna arrays are assumed to be a uniform linear array (ULA) with antenna spacing of *d*_*t*_ and *d*_*r*_, respectively. Both *d*_*t*_ and *d*_*r*_ are small enough so that each individual antenna sees the target at about the same orientation. Typically, *d*_*t*_ and *d*_*r*_ are set to *λ*/2, where *λ* is the carrier wavelength [16]. Each array is divided into *N*_*s*_ subarrays, each of which uses a different orthogonal narrowband signal from a set of waveforms **S** = [**s**_1_^*T*^,…, **s**_*N*_*s*__^*T*^], where each **s** in **S** is a vector of *L* time-samples of the baseband equivalent signals transmitted from each transmit subarray. The number of elements *N*_*s*_ in each subarray need not be identical, although it is assumed to be equal for this analysis. In principle, a subarray can overlap another one [16], but not for the case shown in Figure 1. The transmit antenna array and the receive antenna array are placed on the skin (which is often first lubricated with a special gel) as illustrated in Figure 2, and their positions are close to each other or even on the same location. The *N*_*s*_ waveforms are extracted by a set of matched filters, which gives an output signal vector of length *N*_*s*_ *N*_*r*_. Two distinct characteristics can be observed for this type of system. First, different transmit subarrays each with different orthogonal waveforms result in that the target RADAR cross sections (RCS) become independent random variables. Consequently, a better detection performance can be obtained from the multiple independent measurements. Second, a better spatial resolution can be obtained. In this scenario, the transmit antennas are collocated such that the RCS observed by each transmitting path are identical. The components extracted by the matched filters in each receiving antenna contain information of the transmitting path from one of the transmitting antenna elements to one of the receiving antenna elements. By using the information about all the transmitting paths, a better spatial resolution can be obtained.

A breast or a mammary gland is a highly efficient organ mainly tasked to produce milk and consists of a mass of glandular, chest wall, pectoralis muscles, lobules, nipple, areola, milk duct, fatty tissue, and skin. To simplify the breast structure for wave propagation study, we model it as depicted in Figure 2. As in [17] the Debye model is used to describe the electromagnetic characteristics of the breast tissue,

(1) $$\begin{array}{c} \;\epsilon_{r}=\epsilon_{\infty }+\frac{\Delta \epsilon }{1+\omega \tau }+\frac{\sigma_{s}}{j\omega \epsilon_{0}}, \end{array}$$

where *ϵ*_*∞*_, Δ*ϵ*, *τ*, and *σ*_*s*_ are tissue-dependent parameters. A simpler dielectric model can be used to describe breast tissue such as

(2) $$\begin{array}{c} \epsilon_{r}=\epsilon^{\prime }-j\epsilon^{\prime \prime }=\epsilon^{\prime }-j\frac{\sigma }{\omega \epsilon_{0}}. \end{array}$$

It is assumed that the breast is immersed in a dielectric matching medium, and the skin can be ignored. Normal tissue and tumor tissue are assumed to have the following relative permittivities:

(3) $$\begin{array}{c} \epsilon_{r}^{N}=10\left(1+0.1i\right), \end{array}$$

(4) $$\begin{array}{c} \epsilon_{r}^{T}=50\left(1+0.1i\right), \end{array}$$

respectively [18].

The human body is composed of different organs and tissues, each with different sizes, densities, and corresponding sound velocities; it can then be modeled as an environment with a rich presence of reflectors and scatterers. Consequently, an ultrasonic signal that travels through the breast and is received at the receive ULA can be represented as the sum of numerous attenuated and delayed versions of the transmitted signal, that is, a multipath fading propagation scenario. To characterize the statistical behavior of the received signal, the phase shift of the received signal *ϕ* can be assumed to be uniformly distributed over [0,2*π*] and the magnitude of the received signal *ρ* can be modeled as a Nakagami-distributed random variable [19]. Therefore, the probability density functions of these random variables can be expressed as

(5) $$\begin{array}{c} f\left(\phi \right)=\frac{1}{2\pi }{\text{rect}}_{2\pi }\left(\phi \right),\\ f\left(\rho \right)=\frac{2m^{m}\rho^{2m-1}}{\Gamma \left(m\right)\Omega^{m}}e^{\left(-(m/\Omega )\rho^{2}\right)}U\left(\rho \right), \end{array}$$

where *m* is the Nakagami parameter, *Ω* is a scaling parameter, *U*(·) is the unit-step function, Γ(·) is the gamma function, and rect_2*π*_(·) is the rectangular function of duration 2*π*.

Suppose that there are *K* target points (cancer cells) in the far field at angles *θ*_*k*_, *k* = 1,2,…, *K*, then the corresponding *N*_*t*_ × *K* transmit steering matrix is expressed as **A**(*θ*) = [**a**(*θ*_1_) ⋯ **a**(*θ*_*K*_)], where

(6) $$\begin{array}{c} a\left(\theta_{k}\right)=\left[1\;e^{\left(-j2\pi d_{t}\sin(\theta_{k})/\lambda \right)}\;\cdots \;e^{\left(-j2\pi (N_{t}-1)d_{t}\sin(\theta_{k})/\lambda \right)}\right], \end{array}$$

and the *N*_*r*_ × *K* receive steering matrix **B** is defined in a similar way to **A**. The signal at the receive array can be written as

(7) $$\begin{array}{c} R=\overset{K}{\underset{k=1}{\sum }}B\left(\theta \right)\Gamma A^{T}\left(\theta \right)T^{*}S+W, \end{array}$$

where Γ is a *K* × *K* diagonal matrix of the target complex amplitudes and ∗ denotes the complex conjugate operation. The matrix of size *N*_*t*_ × *N*_*s*_, **T**, has in the *i*th column ones for the elements belonging to subarray *i* and zero otherwise. **W** is the noise component due to sensing error, thermal noise, and clutter returns, which is assumed to be a complex circular symmetric white Gaussian noise with zero mean and covariance matrix **Q**.

After applying a matched filter bank at the receiver, the following output signal vector is obtained:

(8) $$\begin{array}{c} z=Y\left(\theta \right)\gamma +\mathrm{vec}\left(\tilde{W}\right), \end{array}$$

where **Y**(*θ*) is the combined transmit and receive array responses matrix, **γ** is a *K* × 1 vector of the target complex amplitudes, and $\tilde{W}=WS^{H}$ is the noise component of the signal after the match filtering with a covariance matrix $\tilde{Q}=E[\mathrm{vec}(\tilde{W})\mathrm{vec}{\left(\tilde{W}\right)}^{H}]$, where *E*[·] is the expectation operator, *H* is the Hermitian transpose operation, and *vec*⁡(·) is the column-wise stacking operation.

## 3. RADAR Detection Problem

The likelihood function describing the statistical distribution of the received signal after the match filter bank **z** can be expressed as

(9) $$\begin{array}{c} f_{z\;|\;\theta }=\frac{1}{{\left(2\pi \right)}^{N_{z}/2}{\left|\tilde{Q}\right|}^{1/2}}e^{-(1/2){\left(z-Y(\theta )\gamma \right)}^{H}{\tilde{Q}}^{-1}(z-Y(\theta )\gamma )}. \end{array}$$

The radar detection problem is then reduced to the following binary hypothesis test:

(10) $$\begin{array}{c} H_{0}\text{:}\;\;z=\mathrm{vec}\left(\tilde{W}\right)\;\left(\text{a}\;\text{cancer}\;\text{cell}\;\text{is}\;\text{not}\;\text{present}\right),\\ H_{1}\text{:}\;\;Y\left(\theta \right)\gamma +\mathrm{vec}\left(\tilde{W}\right)\;\left(\text{a}\;\text{cancer}\;\text{is}\;\text{present}\right). \end{array}$$

The MAP decision rule can be written as

(11) $$\begin{array}{c} f_{z\;|\;H_{1}}\left(z\;|\;H_{1}\right)p_{1}\overset{\hat{H}=1}{\underset{\hat{H}=0}{⋛}}f_{z\;|\;H_{0}}\left(z\;|\;H_{0}\right)p_{0}, \end{array}$$

where each hypothesis is equally likely; that is, *p*_1_ = *p*_2_ = 1/2. Therefore, the Neyman-Pearson optimal detection rule for this problem is given by the following log-likelihood ratio:

(12) $$\begin{array}{c} L\left(z\right)=\log\frac{f_{z\;|\;H_{1}}\left(z\;|\;H_{1}\right)}{f_{z\;|\;H_{0}}\left(z\;|\;H_{0}\right)}\overset{\hat{H}=1}{\underset{\hat{H}=0}{⋛}}0. \end{array}$$

After some algebraic manipulation, the log-likelihood ration *L*(**z**) becomes

(13) $$\begin{array}{c} L\left(z\right)=-{\left(z-Y(\theta )\gamma \right)}^{H}{\tilde{Q}}^{-1}\left(z-Y\left(\theta \right)\gamma \right). \end{array}$$

Since the targets' amplitudes and directions (or angles) are unknown, their maximum likelihood estimates are estimated as,

(14) $$\begin{array}{c} {\hat{\theta }}_{\text{ML}}=\arg\underset{\theta }{\max}\;z^{H}{\tilde{Q}}^{-1}Y\left(\theta \right){\left(Y{\left(\theta \right)}^{H}{\tilde{Q}}^{-1}Y\left(\theta \right)\right)}^{-1}Y{\left(\theta \right)}^{H}{\tilde{Q}}^{-1}z, \end{array}$$

(15) $$\begin{array}{c} {\hat{\gamma }}_{\text{ML}}={\left(Y{\left(\theta \right)}^{H}{\tilde{Q}}^{-1}Y(\theta )\right)}^{-1}Y{\left(\theta \right)}^{H}{\tilde{Q}}^{-1}z. \end{array}$$

The result from (14) is substituted into (15) to find ${\hat{\gamma }}_{\text{ML}}$. The number of targets (cancer cells) *K* is unknown but they can be estimated using a Bayesian information criterion (BIC) [20]. *K* is estimated minimizing the following BIC cost function:

(16) $$\begin{array}{c} \text{BIC}\left(K\right)=2f\left(\hat{\theta },\hat{\gamma }\right)+3K\ln L, \end{array}$$

where $\hat{\theta }$ and $\hat{\gamma }$ are the estimates of *θ* and *γ*, which are obtained from (14) and (15), respectively. *f* is given by

(17) $$\begin{array}{c} f\left(\theta ,\gamma \right)=L\ln\left|{\left(z-Y\left(\theta \right)\gamma \right)\left(z-Y\left(\theta \right)\gamma \right)}^{H}\right|. \end{array}$$

## 4. Simulation and Results

**Figure 3:**
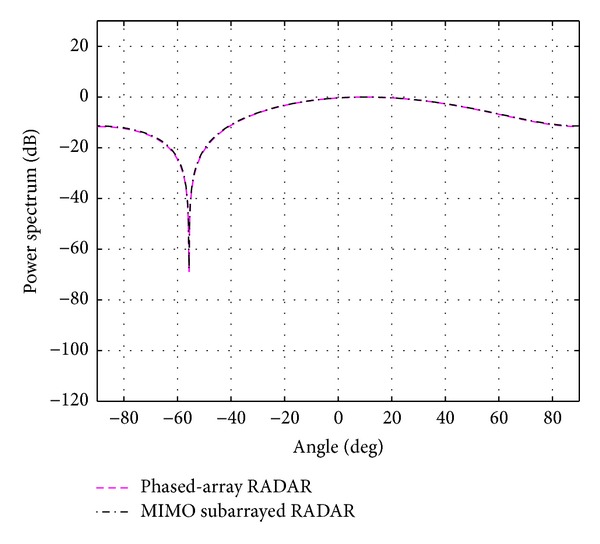
The overall beampattern for [*N*_*t*_, *N*_*r*_, *N*_*s*_] = [2,2, 2].

**Figure 4:**
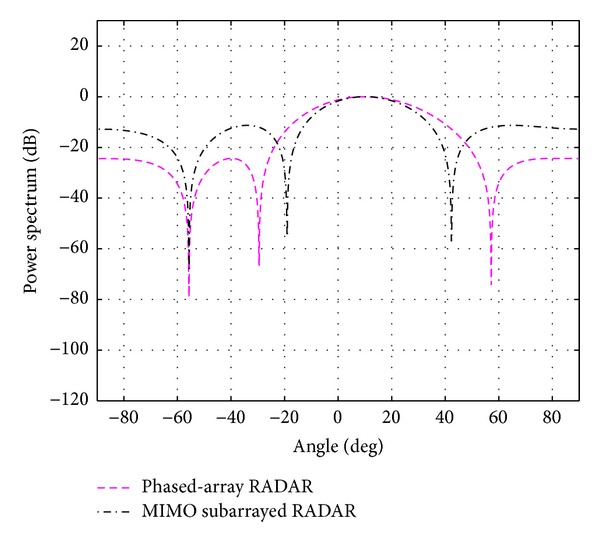
The overall beampattern for [*N*_*t*_, *N*_*r*_, *N*_*s*_] = [4,4, 2].

**Figure 5:**
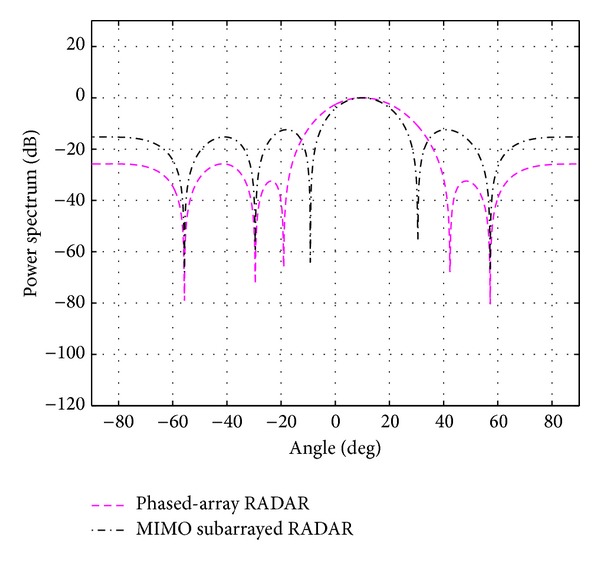
The overall beampattern for [*N*_*t*_, *N*_*r*_, *N*_*s*_] = [6,6, 3].

**Figure 6:**
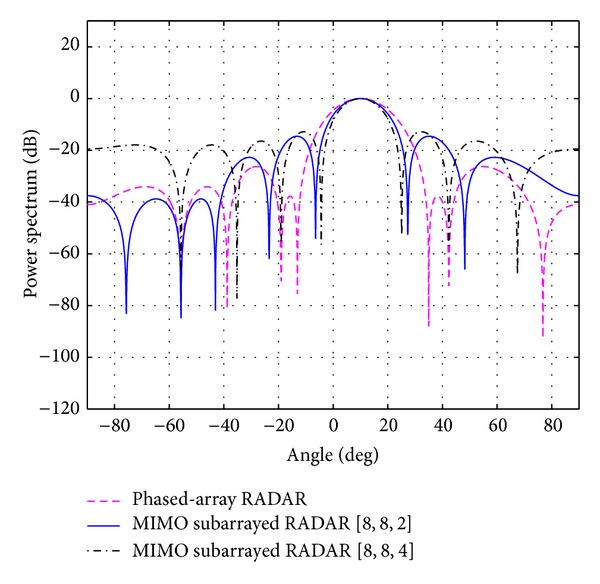
The overall beampattern for [*N*_*t*_, *N*_*r*_, *N*_*s*_] = [8,8, 2] and [8,8, 4].

**Figure 7:**
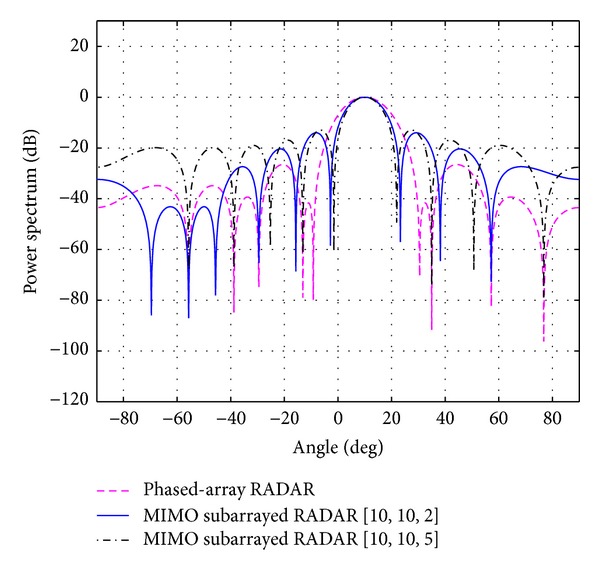
The overall beampattern for [*N*_*t*_, *N*_*r*_, *N*_*s*_] = [10,10,2] and [10,10,5].

The performance of the proposed system is evaluated using Monte-Carlo simulations with 10^6^ runs. All simulation parameters are summarized in Table 1. Current limits imposed by ultrasound safety regulations dictated by the Food and Drug Administration (FDA), an agency of the US federal government, allow for power intensities of up to 720 mW/cm^2^ [21]. Within this limit, a power normalization is carried out so that the target power is fixed to 0 dB. First, it is assumed that there is only one cancer cell position; that is, *K* = 1 in the breast. Using the above mentioned BIC, the number of targets (cancer cells) *K* is estimated to be 1.24, and it is then rounded to 1. The actual target direction with respect to the receive ULA *θ* is set to 10 degrees. Since the carrier frequency applied in the simulations is 15 MHz and the speed of the signal in the breast is assumed to be 1500 m/s [22], the wavelength *λ* becomes 1500/(15 × 10^6^) = 100 *μ*m. The number of antennas in both transmit and receive ULAs *N*_*t*_ × *N*_*r*_ is varied as follows: 2 × 2, 4 × 4, 6 × 6, 8 × 8, and 10 × 10. The number of subarrays *N*_*s*_ for each case is shown in the same table. The overall beampatterns or power spectrums versus angles (which can provide the position of the target using the standard trigonometry expressions) for each case are shown in Figures 3, 4, 5, 6, and 7. It can be observed that the estimation performances of the proposed system are better than the ones from the traditional phased-array RADAR technique since the central lobe is smaller. The beamwidths are reduced when the number of antennas in the ULAs increases meaning that the accuracy of the target position estimate becomes better. In addition, the larger *N*_*s*_ is, the higher the accuracy of the target position estimate is. In practice, the number of antennas in both ULAs cannot be too large. *N*_*t*_ and *N*_*r*_, when more than 10, do not give much different results than when they are 10. Therefore, *N*_*t*_ = *N*_*r*_ = 10 is deemed to provide sufficiently good estimates and it is more practical for an actual implementation. The absolute mean error in degree is summarized in Table 2.

**Figure 8:**
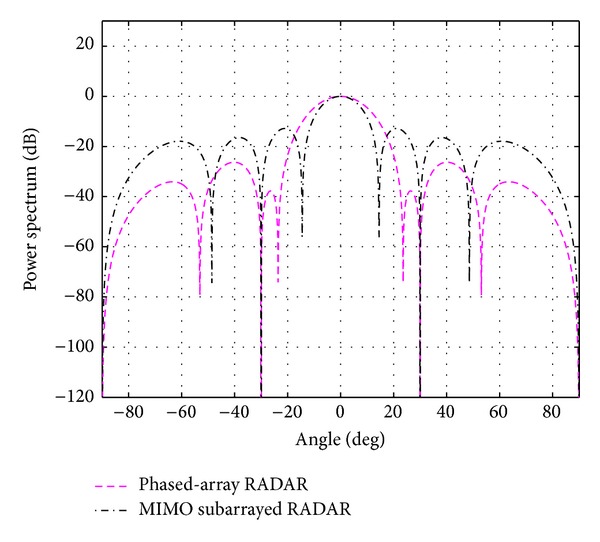
The overall beampattern for two targets at 0 and 20 degrees using *N*_*t*_ = *N*_*r*_ = 8 and *N*_*s*_ = 4.

**Figure 9:**
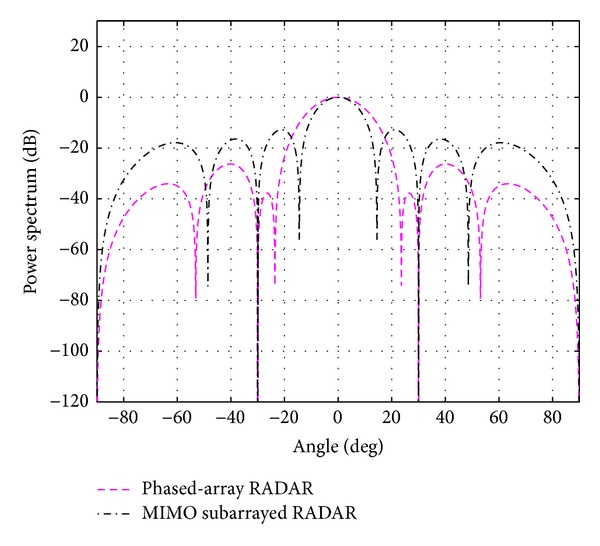
The overall beampattern for three targets at 0, 20, and 40 degrees *N*_*t*_ = *N*_*r*_ = 8 and *N*_*s*_ = 4.

**Figure 10:**
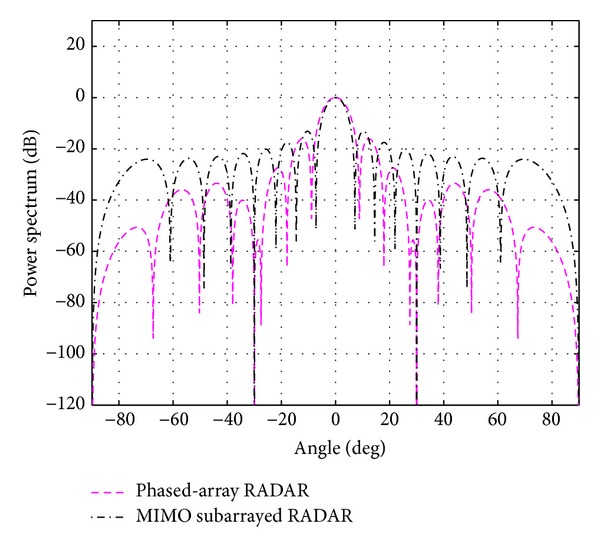
The overall beampattern for two targets at 0 and 10 degrees *N*_*t*_ = *N*_*r*_ = 16 and *N*_*s*_ = 4.

**Figure 11:**
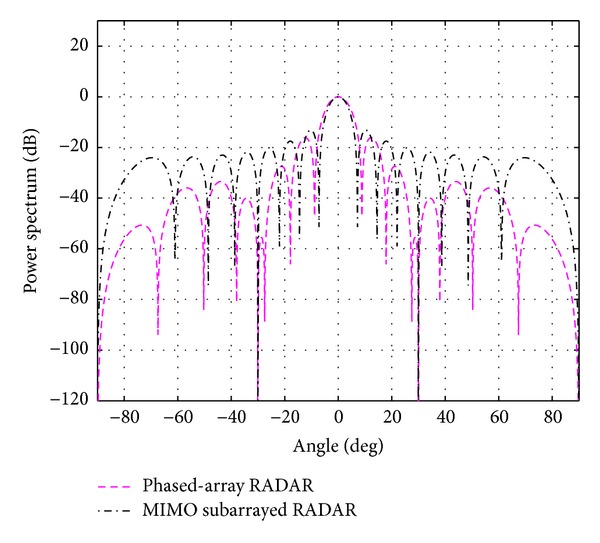
The overall beampattern for three targets at 0, 10, and 20 degrees *N*_*t*_ = *N*_*r*_ = 16 and *N*_*s*_ = 4.

The evaluation is extended for the multiple-target case; that is, *K* = 2 and 3. The case of using *N*_*t*_ = *N*_*r*_ = 8 and *N*_*s*_ = 4 is first considered. With the above mentioned BIC, the number of targets (cancer cells) *K* is estimated to be 2.05 and 3.10 and rounded to 2 and 3, respectively. The simulation results for *K* = 2 at 0 and 20 degrees and for *K* = 3 at 0, 20, and 40 degrees are shown in Figures 8 and 9. Due to the smaller central lobe of the overall beampatterns, the estimation performances of the proposed system are considered to be better than the ones from the traditional phased-array RADAR technique. As it can be seen in Figure 9, it is still possible to use *N*_*t*_ = *N*_*r*_ = 8 and *N*_*s*_ = 4 to successfully detect three targets when they are located 20 degrees apart. However, it is not possible when the targets are located closer, for example, 10 degrees. For this case, more antennas are required. The number of antennas was increased until *N*_*t*_ = *N*_*r*_ = 16 and *N*_*s*_ = 4 were found to be able to detect the targets located 10 degrees apart as shown in Figures 10 and 11. The closer the locations of multiple targets are, the more antennas are needed. The average absolute mean error over for case investigated is summarized in Table 3.

In addition to the system evaluation presented above, a common technique that used for early breast cancer detection is expressed in terms of* sensitivity* and* specificity*.

1. *Sensitivity* is the probability of detecting a cancer if it is actually present in the breast. For example, if 5 women who have cancer are tested and cancer is detected in 4 of them, then the sensitivity is 4/5 or 80%.
2. *Specificity* is the probability of determining that there is no cancer when, in fact, no cancer is present, thereby avoiding a false-positive finding. If 200 women without cancer are examined and 190 are correctly told that they do not have cancer (while the other 10 have further examination), then the specificity is 190/200 or 95%.

**Figure 12:**
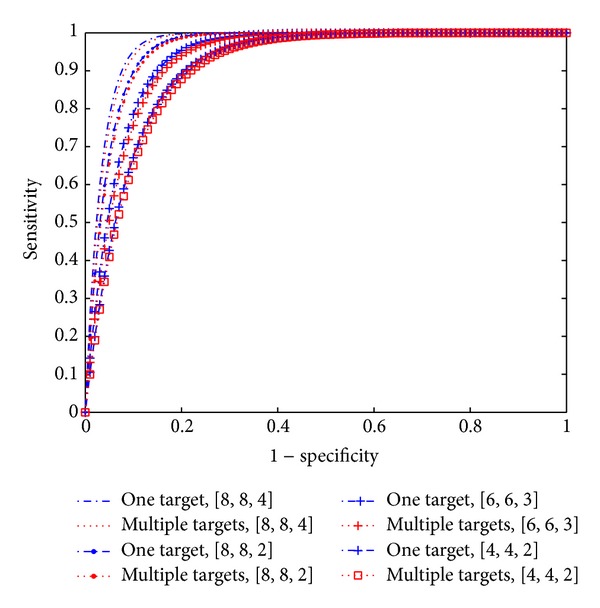
The ROC curve for both one target and multiple target cases.

Most medical tests do not have a single sensitivity or specificity. Instead, these can be varied according to how the test is performed or interpreted. One graphic way of describing the performance characteristics of a test is through what is known as its receiver operating characteristic (ROC), where the sensitivity versus 1-specificity of a test is plotted. A perfect test, represented by the dot in the upper left hand corner of the plot, would find all cancers (100% sensitivity) and create no false positives (100% specificity). To be able to evaluate such a performance, due to lacking of real data and real testbeds, an additional simulation experiment has been carried out. One hundred different types of testbeds are randomly generated with uniform distribution to represent one hundred different testbeds with one target (*K* = 1) (cancer cell) or without cancer. The breasts of each testbed have different sizes and densities, which can be modeled with different Nakagami parameter *m* varying from *m* = 0.5 to *m* = 1 [23]. All one hundred cases are randomly assigned to each testbed. The simulation is run one hundred times (realizations). The result is shown in the ROC curve in Figure 12. The results from another one hundred different testbeds with multiple targets (*K* = 2 and 3) and also without cancer are compared to the one target case. As it can be seen, the ROC performances for the multiple target cases are slightly worse than the ones for the one target cases. Similar to the results of the previously presented detection performance, the ROC performance improves as the number of antennas increases.

## 5. Conclusion and Future Work

In this paper, a subarrayed MIMO RADAR to detect breast cancer position was proposed. The ultrasound frequency band was selected for the proposed system because it is safe and commonly used for medical applications. Since a breast is a superficial structure, high-frequency ultrasonic transducers were used. The transmit and receive antenna arrays were divided into subarrays. Each subarray uses different orthogonal waveforms providing the signal diversity that enables an improved performance. Closed form expressions for the optimal Neyman-Pearson detector were derived. The proposed system combines waveform diversity, made possible by the subarrayed deployment, and traditional phased-array RADAR together with an optimal Neyman-Pearson detection. The system shows promising results for the detection of breast cancer position, not only in the case of one target node, but also in the case of multiple target nodes.

The system described in this paper determines the direction or angle with respect to the ULA where the targets are located. As future work we intend to use a two-dimensional deployment of ULAs so that by properly combining the estimated angles the 3D locations of the targets are estimated.

## Figures and Tables

**Table 1:** Simulation parameters.

Parameters	Values (units)
Monte-Carlo run	1000 (runs)
Carrier frequency	15 (MHz)
Transmit power intensity	700 (mW/cm^2^)
Speed of the ultrasonic signal in breast	1500 (m/s)
Time samples of the baseband equivalent signals *L*	10^6^ (samples)
[*N*_*t*_, *N*_*r*_, *N*_*s*_]	[2,2, 2], [4,4, 2], [6,6, 3], [8,8, 4], [8,8, 2], [10,10,5], [10,10,2]
Number of the targets *K*	1, 2, 3 (points)
Direction of the targets for *K* = 1	10 (degree)
Direction of the targets for *K* = 2	[0,10], [0,20] (degree)
Direction of the targets for *K* = 3	[0,10,20], [0,20,40] (degree)

**Table 2:** Estimation errors for the case of one target *K* = 1.

[N_t_, N_r_, N_s_]	Absolute mean error (degrees)
[2,2, 2]	5.5
[4,4, 2]	2.11
[6,6, 3]	0.53
[8,8, 2]	0.43
[8,8, 4]	0.35
[10,10,2]	0.27
[10,10,5]	0.23
[>10, >10, any number]	about 0.2

**Table 3:** Estimation errors for the case of multiple targets *K* = 2,3.

Number of targets *K*	Average absolute mean error (degrees)
2 at [0,10] degree, *N*_*t*_ = 16, *N*_*r*_ = 16, and *N*_*s*_ = 4	0.33

2 at [0,20] degree, *N*_*t*_ = 8, *N*_*r*_ = 8, and *N*_*s*_ = 4	0.34

3 at [0,10,20] degree, *N*_*t*_ = 16, *N*_*r*_ = 16, and *N*_*s*_ = 4	0.36

3 at [0,20,40] degree, *N*_*t*_ = 8, *N*_*r*_ = 8, and *N*_*s*_ = 4	0.35
